# The Role of Reactive Oxygen Species in Myelofibrosis and Related Neoplasms

**DOI:** 10.1155/2015/648090

**Published:** 2015-10-11

**Authors:** Mads Emil Bjørn, Hans Carl Hasselbalch

**Affiliations:** ^1^Department of Hematology, Roskilde Hospital, Køgevej 7-13, 4000 Roskilde, Denmark; ^2^Institute for Inflammation Research, Department of Rheumatology, Rigshospitalet, Blegdamsvej 9, 2100 Copenhagen, Denmark

## Abstract

Reactive oxygen species (ROS) have been implicated in a wide variety of disorders ranging between traumatic, infectious, inflammatory, and malignant diseases. ROS are involved in inflammation-induced oxidative damage to cellular components including regulatory proteins and DNA. Furthermore, ROS have a major role in carcinogenesis and disease progression in the myeloproliferative neoplasms (MPNs), where the malignant clone itself produces excess of ROS thereby creating a vicious self-perpetuating circle in which ROS activate proinflammatory pathways (NF-*κ*B) which in turn create more ROS. Targeting ROS may be a therapeutic option, which could possibly prevent genomic instability and ultimately myelofibrotic and leukemic transformation. In regard to the potent efficacy of the ROS-scavenger N-acetyl-cysteine (NAC) in decreasing ROS levels, it is intriguing to consider if NAC treatment might benefit patients with MPN. The encouraging results from studies in cystic fibrosis, systemic lupus erythematosus, and chronic obstructive pulmonary disease warrant such studies. In addition, the antioxidative potential of the widely used agents, interferon-alpha2, statins, and JAK inhibitors, should be investigated as well. A combinatorial approach using old agents with anticancer properties together with novel JAK1/2 inhibitors may open a new era for patients with MPNs, the outlook not only being “minimal residual disease” and potential cure but also a marked improvement in inflammation-mediated comorbidities.

## 1. Introduction

The Philadelphia negative chronic myeloproliferative neoplasms (MPNs) encompass essential thrombocythemia (ET), polycythemia vera (PV), and myelofibrosis (MF). These neoplasms arise due to an acquired stem cell lesion with subsequent clonal evolution being driven by several mutations, including the highly prevalent JAK2V617F somatic mutation in PV (in >95%, and in about 50% of patients with ET and PMF, resp.) and the* CALR* and* MPL* somatic mutations [1–9]. These mutations are virtually mutually exclusive and are all considered “second hits” or “driving mutations” within the MPNs whereas the primary genetic hit or “founding mutation” remains unknown [4].

Common clinical denominators for the MPNs are high rates of thrombohemorrhagic complications, hypermetabolic symptoms, splenomegaly, uncontrolled myeloproliferation, low-grade chronic inflammation, a massive inflammation-mediated comorbidity burden, and immune-deregulation [10–16]. The MPNs have an inherent propensity to progress in* a biological continuum* from early cancer stages (ET/PV) to more advanced cancer stages (MF or acute myeloid leukemia (AML)) [17, 18]. The concept of such a* biological continuum* is being increasingly recognized and supported by clinical and molecular studies, the latter displaying increasing JAK2V617F allelic burden throughout the stages. The fact that a* JAK2* positive phenotype only persists in 20–50% of the cases when MPNs transform to AML (or even develops biphenotypic AML) also demonstrates the inherent risk of subclone formation which is a characteristic shared by many other cancers [19–23]. Consequently, the malignant clones are heterogeneous and thus difficult to target with chemotherapy, accounting for the inferior survival in MPN associated AML compared to* de novo* AML [24–26].

The MPNs have recently been described as “*A Human Inflammation Model,”* in which the fuel that feeds the fire is low-grade chronic inflammation [27]. The hypothesis is that the MPN—with uncontrolled myeloproliferation and uncontrolled cytokine secretion as a consequence of constitutively activated JAK-STAT signalling—by itself creates a proinflammatory milieu in the bone marrow and in the circulation. This proinflammatory milieu founds increasing genomic instability accounting for the propensity of the MPNs to acquire new mutations facilitating clonal evolution and ultimately progression to myelofibrosis and AML. It also links the MPNs with a heavy inflammation-mediated comorbidity burden, including premature atherosclerosis, other inflammatory diseases, and second cancers [22, 27–38]. In this context, it has been known for several years that chronic inflammation* per se* increases the risk of cancer development, solid as well as hematological, but the major questions in MPNs are, among others, how low-grade inflammation is eliciting genomic instability and clonal evolution and how the founding clone evades the immune system.

In MPNs, the* optimal therapeutic goals* are to normalize peripheral blood counts, minimize symptoms, prevent vascular complications, restore bone marrow architecture/morphology, and eliminate the risk of progression to MF or evolution to AML. It is crucial to acknowledge that the majority of ET and PV patients have long life-expectancies and therefore treatment related toxicities and long-term side effects influence treatment options [39–42]. The therapeutic agents display striking differences. Treatment with interferon-alpha2 (IFN) has been used successfully for decades, demonstrating its ability to normalize blood counts in the majority of patients, to reduce the JAK2V617F (and* CALR*) allelic burden, and to restore bone marrow morphology and induce major molecular remission in a subset of patients [43–55]. Because of the immune-enhancing properties, some patients experience autoimmune phenomena, primarily thyroiditis, during IFN treatment. A subset of patients also experiences symptoms similar to those arising in patients with systemic inflammation, including chronic fatigue, flue-like symptoms with low-grade fever, weight loss, and depression all symptoms being associated with chronic inflammation [56–58]. Despite undisputed hematological efficacy and safety being shown in a large number of single-arm IFN studies, similar results obtained from large randomized studies between IFN and the most widely used cytoreductive agent in MPNs, hydroxyurea, are still lacking. Most MPN experts agree that HU increases the risk of skin cancer and concern is increasing in regard to its potential of inducing AML after long-term use (>10 years) [59–64]. With the introduction of JAK inhibitors, the therapeutic landscape has expanded considerably. However, these novel agents potently suppress virtually all immune cells including NK-cells, CD4+ T-cells (Th1 and Th17), regulatory T-cells, macrophages, and dendritic cells (DCs) with ensuing impairment of immune regulation and consequently an increased risk of infections [65–71]. This risk is well documented and involves mainly urinary tract infections and herpes zoster but also more rare infections such as tuberculosis, toxoplasmosis, and progressive multifocal leukoencephalopathy [72–76]. Although patients are exposed to an increased risk of infections during treatment with JAK1/2 inhibitors, this novel treatment modality has definitely demonstrated its efficacy in terms of improvement of quality of life due to a rapid resolution of constitutional symptoms within days in concert with a marked reduction in symptomatic splenomegaly within the next weeks or months in the large majority of patients with myelofibrosis [77–80]. To this end, JAK1/2 inhibition in myelofibrosis is associated with an improved overall survival as well [81, 82]. The impact of JAK1/2 inhibition on symptom burden and splenomegaly in myelofibrosis is considered to be driven mainly by its pronounced anti-inflammatory efficacy as evidenced by a marked reduction in several proinflammatory cytokines during JAK-inhibition therapy [77, 83]. In this regard, the improved survival in ruxolitinib-treated MF patients is likely mainly explained by an improvement in inflammation-mediated comorbidities as well [84]. However, ruxolitinib has failed to demonstrate significant impact on the* JAK2* clone [85] which substantiates the need for combinatorial approaches when treating MPNs [28].

Taking into account that chronic inflammation with the production of reactive oxygen species (ROS) may have an important role for the development and progression of MPNs—likely being a very potent driver of clonal evolution and mutagenesis in a vicious self-perpetuating circle—we herein will discuss the role of ROS in MPN pathogenesis and its impact upon comorbidity burden, immune regulation, and disease progression [27, 29, 86–90].

## 2. Reactive Oxygen Species

Reactive oxygen species (ROS) are a group of oxygen-containing molecules involved in many biological processes including normal cellular signalling and immune defence. Consequently, lacking the ability to produce ROS results in organ dysfunction and disease as evidenced by, for example, chronic granulomatous disease in which the immune system is unable to combat invading bacteria and fungi due to impaired production of ROS by neutrophils [91–95]. However, the same ROS compounds are also involved in several inflammation-driven diseases where an excess of ROS production is thought to account for the tissue damage, dysfunction, and fibrosis associated with the diseases [96, 97]. In addition, elevated levels of ROS, often referred to as* oxidative stress*, have a major role in cancer development, both in solid tumors and in hematological malignancies [86–90, 97]. There is no clear cut-off that defines exactly which compounds are to be included in the ROS category, and often nonoxygen molecules buffering ROS levels are also included in the analysis of cellular oxidative status. The molecules superoxide (O_2_^−^) and hydrogen-peroxide (H_2_O_2_) are obvious ROS, but intracellular levels of glutathione and reduced glutathione are also crucial in the cellular redox interplay. Hydrogen-peroxide is of particular interest since it can freely diffuse across cellular membranes and interact with cells in close proximity to the H_2_O_2_ producing cells. This includes the endothelial cells within the intima of artery walls, and oxidative stress has already been linked to cardiovascular diseases, especially the development of premature atherosclerosis in chronic inflammatory diseases [96, 98–100]. H_2_O_2_ has been shown to activate NF-*κ*B pathway, thus creating self-perpetuating vicious circles in which inflammation creates ROS which in turn creates more inflammation [101–103]. To avoid such situations, the system has a fail-safe:* suppressors of cytokine signalling* (SOCS), a family of proteins dedicated to creating negative feedback loops. They are normally activated by inflammatory mediators such as IFN, IL-4, TNF-alpha, and H_2_O_2_ [104, 105]. Activated SOCS proteins bind to JAKs disrupting the JAK-STAT pathway, thereby ensuring that the inflammatory process is not being sustained. However, in MPNs, this pathway is constitutively activated and the much warranted SOCS brake is overruled. Furthermore, aberrant methylation of SOCS-coding DNA and consequent dysregulation of SOCS have also been reported in MPNs [106, 107].

## 3. Hepatitis C as a Model of Inflammation-Mediated Fibrosis and Cancer Development: Similarities to MPNs as “A Human Inflammation Model for Cancer Development”

The initiating event in hepatitis C is a viral infection. This results in chronic inflammation, increased production of ROS and consequently oxidative stress, inability of the immune system to clear the infected cells, an increased risk of progression to terminal cirrhosis, and ultimately an increased risk of developing hepatocellular carcinoma (HCC) or lymphoma [108–113]. In MPNs, the initiating event is unknown, but after acquisition of the* JAK2* mutation, MPNs (much like hepatitis patients) exhibit evidence of low-grade chronic inflammation with ensuing fibrosis and bone marrow failure in addition to an increased risk of developing AML. Another similarity is the inability of the host immune system to identify and clear the fundamental problem, for example, the malignant clone in regard to MPNs. Another striking similarity is the existence of a common effective treatment modality: the very potent immune-enhancing, antiviral agent IFN which has been used successfully for decades in hepatitis patients as well as in MPN patients. In this regard, it has recently been hypothesized that a virus infection (e.g., human retrovirus) might be implicated in MPN pathogenesis [27, 114]. It is also of particular interest to note that oxidative stress has been implicated in the therapeutic response. Thus, it was demonstrated, that increasing levels of ROS disrupt IFN signalling, thus counteracting therapy [115].

## 4. ROS and MPNs

**Figure 1:**
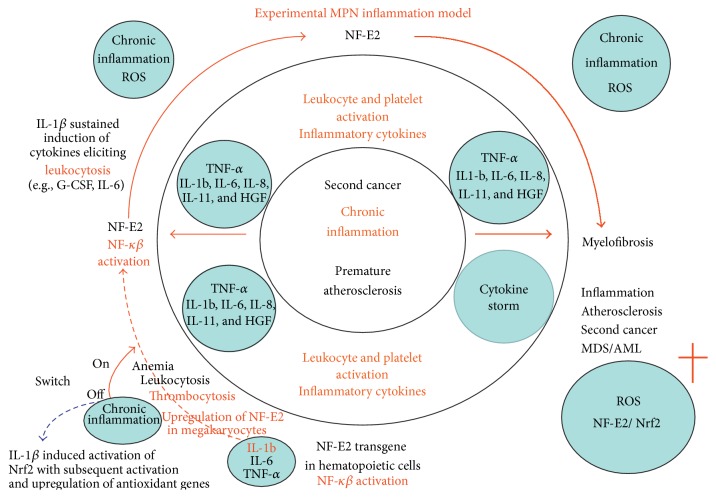
Sustained NF-E2 expression likely elicits a pronounced oxidative stress milieu with excessive ROS giving rise to myeloid expansion with leukocytosis and excessive thrombocytosis and inflammation-mediated* in vivo* activation of leukocytes and platelets, thereby further promoting a sustained, self-perpetuating release of inflammatory products. In this vicious circle, an oxidative stress burden with NF-E2 domination over Nrf2 promotes ROS accumulation and megakaryocytic differentiation. Increasing oxidative stress-induced DNA damage of hematopoietic stem cells (HSCs) elicits genomic instability and clonal MPN evolution with accumulation of mutations ultimately terminating in myelofibrotic and leukemic transformation. A relative deficiency of Nrf2 may also result in expansion of the HSC and progenitor cell compartment and ultimately migration of HSCs from their stem cell niches into the circulation (“leaving the burning nest”) to seed in the spleen and liver (myelofibrosis with myeloid metaplasia). The vicious circle may be locked by early intervention with interferon-alpha2 (stopping the fuel to the fire) in combination with a JAK1-2 inhibitor (e.g., ruxolitinib) and a statin, the latter agents “cooling down the system” by their highly potent anti-inflammatory properties which may actually be enhanced (synergism) when being administered simultaneously. With permission from Leukemia Research [29].

The ROS molecules are produced mainly by neutrophils, macrophages, and monocytes. In the context of MPNs, this is crucial, since the MPN cells are clonal and autonomously dysregulated and have been shown to produce excessive ROS* in vitro* [87]. Furthermore, MPN patients demonstrate elevated levels of ROS* in vivo* [86, 116]. An increased ROS production has been observed in other cancers, and in some cases the cancer cells express catalase (the enzyme that metabolizes H_2_O_2_) in excess and in addition produce large amounts of H_2_O_2_. In this way, the malignant clone itself avoids the toxic effects of H_2_O_2_ and suppresses the neighbouring healthy cells (ROS induce apoptosis in healthy cells) thereby facilitating clonal expansion [117–126]. This mechanism has not yet been established in MPNs but certainly warrants further investigation, especially since the excessive ROS production in MPNs gives rise to a proliferative advantage to* JAK2* positive clones [28, 87, 127, 128] (Figure 1). In this regard, the model proposed by Marty et al. is in agreement with the MPN inflammation model and excessive ROS accumulation in a vicious self-perpetuating circle. In this context, considering the role of NF-E2 in MPN disease pathogenesis, it is intriguing to speculate if NF-E2 may contribute in driving the vicious inflammation wheel, including ROS accumulation as most recently discussed [29, 129–134]. On the other hand, it has also been demonstrated that the hematopoietic stem cell niche (HSC) in MPNs downregulates catalase activity resulting in an increase in oxidative DNA damage (8-oxo-G) and subsequent double-stranded- (ds-) DNA breaks, a widely accepted measure of ROS induced DNA damage, and perhaps in this way induces instability of the HSC niche [87].

In a mouse model, ds-DNA breaks were shown to be a consequence of ROS accumulation, and it was also shown that the CD34+ HSCs themselves produced this excess ROS, probably as a consequence of catalase downregulation [87, 127]. Furthermore, a functional lack of superoxide dismutase (SOD) activity could also be of importance. ROS negatively influence the AKT pathway, which in turn influences Forkhead O/FoxO which regulates the transcription of several antioxidative defence pathways, including GPx, catalase, and SOD [135]. These mice developed aggressive PV phenotype but when they were treated with the potent ROS-scavenger molecule n-acetyl-cysteine (NAC) they developed normal phenotype, demonstrating the direct role in MPN disease development and disease progression [87]. This was substantiated by the finding that NAC treatment of the PV phenotype mice delayed progression to MF phenotype when compared to nontreated mice.

The damaging effects of ROS (besides the proliferative advantage) are also attributed to the consequent oxidation of lipids, proteins, and, most importantly, the ds-DNA breaks due to oxidation. In healthy cells, this insult will be rapidly repaired but a hallmark of most cancers is a defective DNA repair (sometimes even induced by therapy, e.g., hydroxyurea). Furthermore, the response to DNA damage is also affected as demonstrated by the negatively regulated p53 pathway in MPNs [136]. This is also demonstrated in MPNs, where the CHEK2 germline mutations, which are associated with ET and PV, account for an increased risk of developing an MPN. Together with other proteins, the CHEK2 proteins are associated with DNA damage and binding of TP53 (p53) and CHEK2 are involved in many cancers [137–143]. Consequently, harbouring this CHEK2 mutation can result in inadequate DNA repair and consequently increased risk of developing (and sustaining) genetic hits in several cancer types. It is intriguing to consider if germline CHEK2 mutation accounts for the initial genetic instability in some MPN patients. In time and by “chance” this might result in a somatic* JAK2* mutation with ensuing increased production of ROS, clonal expansion, and increasing genomic instability due to ineffective DNA repair and an increase in ROS induced DNA damage, all of which further facilitate disease progression with subclone formation and inflammation-mediated bone marrow fibrosis. The role of chronic inflammation and ROS in MPN pathogenesis has most recently been substantiated in a mouse model, in which mice were exposed to the highly potent inflammatory compound, formaldehyde (FA), by inhalation [144]. This agent induced bone marrow toxicity with typical MPN-like alterations in the mice, including an increased number of megakaryocytes and myelofibrosis in concert with the development of anemia, leukopenia, and thrombocythemia. Highly interestingly, these changes were accompanied by evidence of oxidative stress and inflammation in the bone marrow as assessed by significant increases in ROS levels, increased NF-*κ*B activity at both mRNA and protein levels, and significant increases in the inflammatory markers, TNF-alpha and IL-1beta, as well [144]. These observations are in accordance with studies demonstrating that oxidative stress in hematopoietic stem cells can lead to DNA damage, premature senescence, and loss of stem cell function [145]. Accordingly, all together these findings are supportive of the concept that chronic inflammation by induction of oxidative stress and an inflammatory bone marrow microenvironment may give rise to DNA damage and likely an impaired stem cell function with ensuing development of myelofibrosis.

In hepatitis, it has been demonstrated, that the excess of ROS and consequent* oxidative stress* inhibits IFN signalling, thus counteracting the normal immunosurveillance by NK-cells and CD8+ cytotoxic T-cells (CTLs) [115]. The reduced IFN signalling and ensuing reduction of MHC-I expression by virally infected cells provide an escape route from the innate and adaptive immunosurveillance. A prerequisite of this model is that the MHC-I expression is low enough to avoid CTL activation by antigen recognition, but also high enough to avoid “missing self” activation of NK-cells. This model deserves to be tested in MPNs to elucidate if increased ROS levels might facilitate both clonal expansion and immune evasion, implying ROS-mediated inhibition of IFN signalling and the immune evasion to exhibit dual actions. In this regard, reduced MHC-I expression might keep the tumor below detection limit of CTLs, and even* if* a tumor cell is indeed detected, probably due to threshold expression of “self” by MHC-I, the consequent IFN signalling from the activated NK-cell will likely have only a limited impact since the pathway is functionally blocked by excess of ROS. By this mechanism, recruitment and activation of other immune cells, in particular macrophages, may be inhibited and the immune response remains unamplified and thus ineffective in combating the clone. This concept is partly supported by the finding of downregulation of HLA expression in ET, PV, and MF and further supported by efficacious treatment with IFN, which increases MHC-I expression of the clonal cells (thus making them “legitimate” targets for CTLs) but also increases the NK-cell compartment, thus inducing the much warranted amplification of the immune system. IFN also mobilizes dormant stem cells rendering them susceptible to targeted therapies [146–149].

Since ROS appear to play a crucial role in disease progression of MPNs, the targeting of ROS seems intuitive, especially since the increased ROS can interfere with both endogenous tumor surveillance and treatment response. Treatment with NAC has been used successfully in an* in vivo* mouse model after JAK2V617F bone marrow transplant, but never in human MPN subjects. The majority of experiences with human NAC treatment are based on the treatment of patients suffering from paracetamol poisoning. In this setting, the NAC treatment is intensive and of short duration. NAC treatment has also been investigated in spinal cord injuries but again the treatment duration is short [150–153]. However, longer exposure has been investigated in chronic pulmonary diseases: chronic obstructive pulmonary disease (COPD) and cystic fibrosis (CF). Both diseases have a significant inflammatory component, and both diseases showed positive responses to NAC treatment with fewer exacerbations (COPD) and more stable lung function (CF) in the NAC-treated groups [154–156]. Similar results were obtained in patients suffering from systemic lupus erythematosus (SLE) [157].

In order to target MPN related oxidative stress, it is important to acknowledge that the optimal level of ROS is not known. In the experimental MPN models, treatment with NAC almost totally removed any existing ROS, which in an experimental model might give satisfying results but in a human trial might result in a dismal outcome [87, 127].* In vivo*, ROS are needed to some extent to ensure normal cellular signalling and to enable the immune system in combating invading bacteria and an obvious problem might be an increase in infectious diseases and (other) neoplastic diseases. However, this has not been identified so far with NAC treatment of COPD and CF, both diseases otherwise heavily burdened by (chronic) infections and the NAC treatment resulted in disease relevant improvements.

## 5. Discussion and Perspectives

The MPNs are clonal neoplasms intimately associated with a dysregulated immune system [16, 17, 148, 149, 158–160]. As in many other diseases, inflammation and excess generation of ROS are thought to play a major role, both in disease initiation and associated inflammation-mediated comorbidities [27, 28, 84, 135]. The initiation of disease is probably a consequence of defective DNA repair and/or increased acquisition of DNA damage. This could be caused by many factors, for example, germline CHEK2 mutation [142, 143]. Of note, it is also intriguing to consider that the initiation of the disease might be consequent to a “fertile ground” changing the fitness of the stem cell niche for a preexisting abnormal hematopoietic stem cell [4, 161–163]. By chance, the JAK2 mutation is acquired and consequent generation of ROS with clonal expansion and evolution due to genomic instability characterizes the further course of the disease. ROS are also involved in cardiovascular diseases which are major contributors to the comorbidity burden and mortality in MPN patients [11, 15, 96, 98, 99]. Accordingly, the targeting of ROS is an obvious therapeutic option, especially since one of the main goals is to prevent genomic instability, likely facilitated by increased ROS, and thereby ultimately fibrotic and leukemic transformation. In regard to the potent efficacy of NAC in decreasing ROS levels, it is intriguing to consider if NAC treatment might benefit patients with MPNs. The encouraging results from studies in CF, SLE, and COPD warrant such studies.

Furthermore, the antioxidative potential of the widely used agents, IFN, JAK inhibitors, and statins, both as monotherapies and in various combinations, should be investigated. Studies on combinations with IFN, the only agent with the potential to induce “minimal residual disease,” as the* backbone* and “old antioxidative drugs” (statins, NAC) and the novel JAK1/2-inhibitors are urgently needed. Such studies may further enhance the potency of the novel combination therapy with IFN and ruxolitinib, a concept which already has been shown to be highly efficacious in patients with PV and hyperproliferative MF, implying an improvement in inflammation-mediated comorbidities as well [28, 84, 164]. Such a combinatorial approach using old agents (statins, NAC, and IFN) with anticancer properties (antiproliferative, proapoptotic, antiangiogenic, anti-inflammatory, and antioxidative properties) together with novel JAK1/2 inhibitors may open a new era for patients with MPNs, the outlook not only being “minimal residual disease” and potential cure but also a marked improvement in inflammation-mediated comorbidities. These goals will not only set new standards for treatment of MPNs in the future but may also likely be highly cost-effective when considering the potential of decreasing dosages of very expensive drugs (JAK1/2 inhibitors/ IFN) due to synergism between them and for example, statins, and therefore a reduction in side effects of single agents as well [27, 28, 165–168]. This novel treatment concept, targeting the oxidative stress mechanisms in MPNs, is foreseen to alleviate the heavy disease burden, which encompasses not only an increased risk of severe cardiovascular complications and second cancers but likely also an increased risk of premature atherosclerosis (early ageing?) [28, 29, 135]. By eliminating the oxidative stress overload, improving the defective antioxidative stress defence, and improving “Tumor Immune Surveillance” according to the novel treatment concept as outlined above, we are convinced that the future will look bright for our patients and will enlighten new horizons.
